# A feeling of not being alone – Patients' with COPD experiences of a group‐based self‐management education with a digital website: A qualitative study

**DOI:** 10.1002/nop2.2153

**Published:** 2024-04-19

**Authors:** Mia Berglund, Susanne Andersson, Anna Kjellsdotter

**Affiliations:** ^1^ School of Health Sciences Skövde University Skövde Sweden; ^2^ FoU‐centrum Skaraborg ‐ R&D Centre Skaraborg Region Västra Götaland Skövde Sweden; ^3^ Department of Health Sciences University West Trollhättan Sweden; ^4^ Research and Development Centre Skaraborg Hospital Skövde Sweden

**Keywords:** COPD team, eHealth, patient involvement, phenomenology, self‐management, sustainable learning

## Abstract

**Aim:**

To describe patients' with chronic obstructive pulmonary disease (COPD) experiences of group‐based self‐management education with a digital website.

**Design:**

A qualitative approach with a phenomenologicalmethod. Patients participating in an earlier study, with self‐experience of COPD as a special competence, were involved as research partners at the design of this study.

**Methods:**

Eleven individual and two group interviews with five participants in each group were conducted.

**Results:**

Group‐based self‐management education with a digital website supports learning. Sharing experiences with others in similar situations creates security and reduces the feeling of being alone. Based on questions and discussion in the group, and through self‐reflection, general information is transformed into useful knowledge and understanding of one's own situation. COPD information on the website provides an opportunity to gain knowledge continuously based on needs that contributes to learning. This research has demonstrated that adapting learning activities to individual learning styles increases sustainability of learning. Sharing experiences reduces feelings of loneliness. It is therefore important to create spaces for sharing experiences and in‐depth reflection that support learning over time.

## 1 INTRODUCTION

Chronic obstructive pulmonary disease (COPD) is a major cause of chronic morbidity and mortality, and with increasing prevalence, the disease will rank third in world mortality by 2030 (World Health Organization, 2020). In Sweden, it is estimated that 500,000–700,000 people have been diagnosed with COPD at present (The National Board of Health and Welfare, 2018). COPD is not fully reversible with treatment due to permanent lung damage but its progression can be slowed through, for example, self‐management activities (McCarthy et al., 2015). The illness is progressive and associated with considerable disability, impaired quality of life, and high utilisation of healthcare resources (McCarthy et al., 2015; Stellefson et al., 2018). COPD is often associated with statistically significant co‐morbidities including cardiovascular diseases, diabetes, hypertension, depression, and others (Mannino et al., 2008; Yohannes & Alexopoulsm, 2014). The goal of all COPD treatment is to help patients control their illness as much as possible.

Given this goal, COPD treatment should be person‐centred, focusing on the patient as an active partner in care and treatment (Ekman et al., 2011) and integrating their experiences, needs, resources, and condition. Person‐centred patient education, smoking cessation, physical exercise, and multidisciplinary self‐management support are essential in the treatment of COPD (Kaptein et al., 2014; Stoilkova‐Hartmann et al., 2018). It is important to strengthen the patient's motivation to practice self‐management which will help them best manage their illness in daily life and make them more likely to seek appropriate and timely care (Kaptein et al., 2014; Newman et al., 2004).

Self‐management education is a high priority in the Swedish National Guidelines for Asthma and COPD [2] (The National Board of Health and Welfare, 2018). Research has shown that the care of persons with COPD is more impactful when based on multidisciplinary collaboration and consists of self‐management education in COPD rather than traditional biomedical education classes (Kruis et al., 2013). However, it is important that such rather than just information to optimise quality of life, increase independence, maintain a stable health condition as long as possible, and prevent periods of exacerbation in patients living with COPD (Kruis et al., 2013; Stoilkova‐Hartmann et al., 2018). COPD self‐management education should therefore comprise a person‐centred approach as it emphasises patients' needs and sharing experiences in group with other patients and professionals (Ali et al., 2018). In line with person‐centred care, health professionals need to increase patient involvement, which in turn implies a need to change routines and establish new ways of organising and administering care. Digital solutions, such as eHealth interventions, seem to facilitate person‐centred care, strengthen patients' position in the health service system, and support their self‐management strategies in everyday life (Barenfeld et al., 2020; Tistad et al., 2018). The current evidence around eHealth interventions in chronic disease management is growing, and established digital tools have already shown to effectively support self‐management in patients with long‐term conditions. However, effects are inconsistent and further research is needed (Hanlon et al., 2017). Therefore, this study aimed to describe patients' with COPD experiences of a group‐based self‐management education with a digital website.

## 2 METHODS

### 2.1 Study design

A qualitative research design was conducted, with an open and reflective attitude, to explore and illuminate the phenomenon “group‐based self‐management education with a digital website,” throughout the research process (planning, organising, and analysing data). The reflective life‐world research (RLR), based on phenomenological philosophy, is developed by Dahlberg et al. (2008). Life‐world is understood as the everyday world of experiences. It is usually un‐reflected and taken for granted in daily life. However, through reflection experiences can become aware, conceptualised and possible to share.

Phenomenological research focus on how people experience the phenomenon of interest, how it can be described and understood. The transferability of the findings may be possible if a rich variety of experiences forms the basis for phenomenon‐oriented essence‐seeking analysis, which contribute to increased knowledge of importance for similar patient groups with long‐term illnesses and high self‐care ability (Dahlberg et al., 2008; Dahlberg & Dahlberg, 2020). For reporting qualitative research, we applied the Standards for Reporting Qualitative Research (SRQR) guidelines (O'Brien et al., 2014).

### 2.2 A multidisciplinary team and patient involvement in research

The COPD team at the hospital consisted of fourteen professionals with extensive experience in caring for patients with COPD. The COPD team and the group of eight patients with self‐experience of COPD (both men and women) were actively involved in selected parts of the research process. Patient involvement in research was developed and strengthened with the help of a workshop followed by regular meetings (Bate & Robert, 2006; Boyd et al., 2012). The scientific issues were discussed; study information and an interview guide were worked out through continuous feedback by mutual reflection and action between the researchers, the COPD team, and the involved patients as a guiding principle. The participants involved as partners in research should be able to represent a broader perspective than their own (Liabo et al., 2018). These patients are regarded as representatives of the group of persons living with COPD, experts on their own and others' situation and conditions.

### 2.3 COPD school with a digital tool (COPD website)

Patients living in the region of the study hospital, the south‐west part of Sweden, are offered a structured education in line with the Swedish National Board of Health's guidelines for asthma and COPD (The National Board of Health and Welfare, 2018). The group‐based self‐care education (COPD school) was divided into different educational blocks comprised of four occasions à 2 h each (Table 1). Group size usually varied between 8 and 10 persons with COPD and their relatives to strengthen each group's dynamics.

**TABLE 1:** Group session themes at the COPD school.

Session	Profession	Responsibility
1 COPD treatment, medication and psychosocial support, introduction to the COPD website	COPD nurse^a^	Lifestyle changes, examination with spirometry
	Physician^a^	Facts about COPD
	Pharmacist^a^	COPD drugs, self‐care at mild deterioration, vaccinations
	Speech therapist^a^	Speaking exercises, swallowing difficulties
	Medical counsellor^a^	Information on patient rights in hospitals and in society; emotional, informative, financial and practical support
	The local patient organisation HeartLung	A representant from the organisation HeartLung
2 The importance of food to COPD, oral health, and energy‐saving methods in daily life	Dietician^a^	Dietary guidelines
	Dental hygienist^a^	Oral health; information about economic dental grant
	Occupational therapist^a^	Utilities in everyday life, energy‐saving methods
3 Self‐care, breathing, and coughing techniques and physical activity	Physiotherapist^a^	Breathing and coughing techniques, relaxation, energy‐saving methods, mobility, strength and stretching
4 Physical exercise including pelvic floor exercises	Physiotherapist^a^ Urotherapist^a^	Practical exercises

^a^ Education at the COPD website.

**FIGURE 1:**
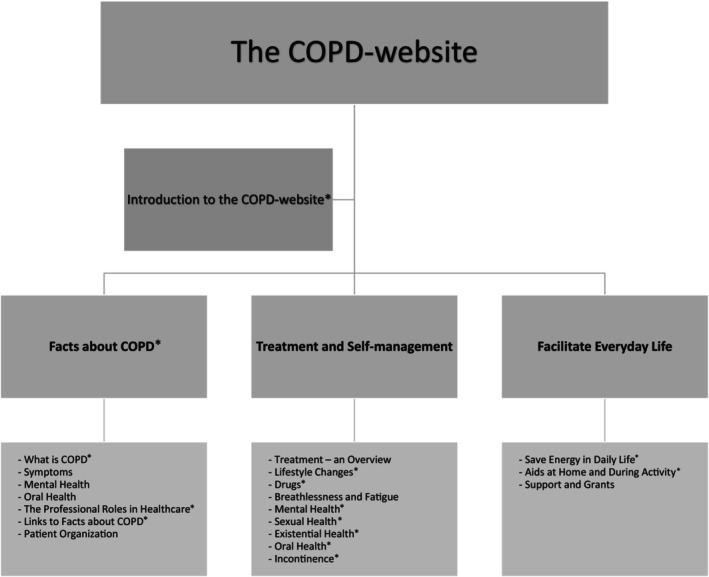
In the image above all themes contain factual texts and pictures, *also contain films. Some themes are exemplified by subthemes.

The COPD website, as a complement to the COPD school, was created for future needs by health professionals in collaboration with patients with self‐experience of COPD. It was made self‐instructional and divided into three main themes: *Facts about COPD, Self‐management and treatment, and Facilitating everyday life* (Figure 1) (Kjellsdotter et al., 2021). Under each theme, there were several sections containing one or more texts, pictures, and films. Exploring patients' experiences will enhance our understanding of self‐management in patients with COPD and help identify potential targets for improving self‐management support.

### 2.4 Ethical considerations

The study conformed to the principles of the Declaration of Helsinki (WMA, 2008). Verbal and written informed consent were obtained from all participants. The study was approved by the National Ethical Review board (Dnr: 2019‐02293). The Standards for Reporting Qualitative Research (SRQR; Appendix S1) were used to guide reporting of data (O'Brien et al., 2014).

### 2.5 Data collection

#### 2.5.1 Setting and participants

This study's participants were patients with experience of living with COPD recruited from a central hospital in the south‐west region of Sweden. Participants in the COPD school with a digital tool were asked to participate in the study. The study included 21 participants, 20 patients with COPD and one relative, nine men and 12 women in the age range of 61–84 years who had participated in the COPD school. No exclusion criteria were used.

#### 2.5.2 Interviews

Data collection consisted of interviews with the aim of obtaining as rich descriptions of meaning as possible of the studied phenomenon, which according to Dahlberg and Dahlberg (2019) is of crucial importance to the quality of qualitative research.

To get as rich descriptions of meaning as possible, both group interviews and individual interviews were conducted. During group interviews, informants are encouraged to share experiences, discuss, and reflect on the phenomenon of interest. During individual interviews, the informant, with support of questions, conceptualises their individual and un‐reflected experiences. In this way, the researchers are given access to lived experience on both group and individual level to deepen the understanding of the phenomenon (Dahlberg et al., 2008).

Two group interviews (with five participants in each group) were conducted by two researchers (AK, SA) in a conducive environment in separate rooms at the hospital. The group interviews were carried out to enable and ensure that a dialogue occurred among the informants.

Eleven individual interviews were carried out as a dialogue to enable reflection on the personal experiences of the phenomenon of interest. The individual interviews were performed by all authors (AK, MB, SA) and were conducted over the telephone, as chosen by the informants due to the COVID pandemic.

The initial questions in both groups and individual interviews were as follows: Please, can you tell us about your experiences concerning participation in group‐based education (COPDschool)? Please, describe how you used the digital self‐care education (the COPD‐website)? Utilised open‐ended and follow‐up questions as: Please, tell me more? Can you give examples? were also asked to capture personal experiences (Dahlberg et al., 2008).

Each group interview lasted about 75 min, while the individual interviews lasted 20–37 min. The interviews were digitally recorded and transcribed verbatim and anonymised.

### 2.6 Data analysis

Phenomenological research requires an open and sensitive attitude, and the researchers involved must bridle their understanding throughout the research process. According to Dahlberg et al. (2008), bridling requires a critical and reflective attitude. As human beings, we relate to meaning constantly. The challenge with a bridling attitude is to be reflective, careful and open for nuance of the meanings, to have the ability to slow down the understanding process, that is, not understand too quickly (Dahlberg & Dahlberg, 2019).

All collected data were seen as one whole piece of text. In the first analysis phase, the text was read in an open manner to gain familiarity with the data. The data were then divided into sequences (units) with their own meaning. The meaning units were further reflected against the whole data. In the next phase, groups of meaning were built up as clusters. In the cluster analysis, a pattern emerged, and the essence formed. This can be described as an abstract synthesis of the phenomenon's unique structure of meanings, which creates a new whole (Dahlberg et al., 2008); on a more concrete level. The phenomenon becomes nuances or constituents illustrated with interview quotes. Throughout the process, there was a movement between the whole and the parts to create a new whole. Through a creative and critical reflection, the clustering, and the creation of the essence with the constituents took place. During the process of projecting the result, one researcher (MB) had responsibility for the whole and the others (AK, SA) read with a critical bridled approach to maintain openness to the phenomenon. The trustworthiness was secured throughout the analysis when the authors repeatedly discussed the essence and the constituents, to reach a final consensus, this is in line with (Dahlberg & Dahlberg, 2020).

**FIGURE 2:**
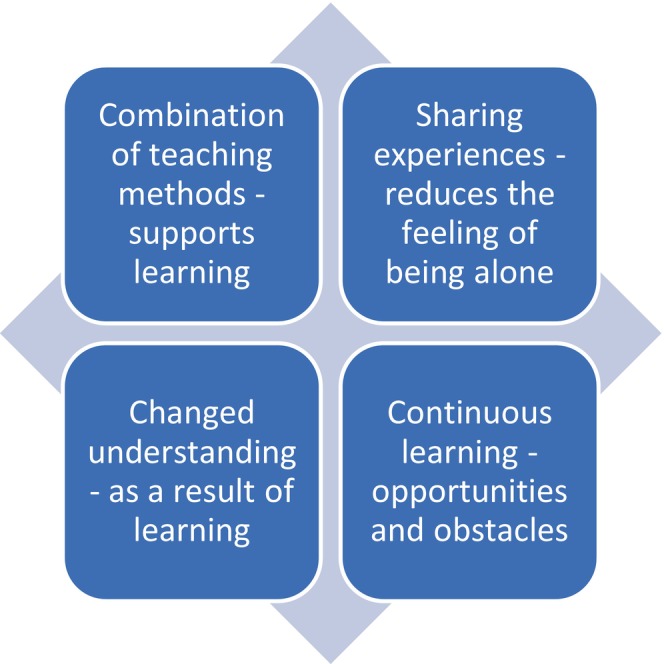
The essence of the phenomenon group‐based self‐management education with a digital website with the four constituents.

In this paper, the essence is presented in the results, followed by the four constituent elements of the phenomenon (Figure 2), which are illustrated with quotations from the interviews. The group interviews are labelled G1 and G2 and the individual P3–P13 individual interviews.

## 3 RESULTS

The essence of the phenomenon of group‐based self‐management education with a digital website as experienced by patients with COPD is that the combination of seeing, listening, and doing, both physically and digitally, support learning. Sharing experiences and feelings, such as shame and guilt over being affected by COPD and worries about the future, with others in similar situations creates a sense of security and reduces the feeling of being alone. Based on questions and discussion in the group, and through self‐reflection, general information is transformed into useful knowledge and understanding of one's own situation. COPD information on the website provides opportunities to gain knowledge before, during, and after the group training sessions and contributes to continued learning both for the patients and for their family members.

### 3.1 Combination of teaching methods – Supports learning

Group‐based self‐care education with digital support aimed for persons with COPD entails a combination of different teaching methods, such as lectures, films, and/or practical demonstrations. It also involves a combination of formal and informal learning. This combination supports learning that improves the safety and well‐being of patients living with COPD.

The lectures provide information about the disease, its causes, and possible deterioration as well as self‐care advice related to diet, physical activity, and drug administration. Participants appreciate that the physical group sessions and the information available on the website cover the whole body: “from the mouth down” (GP2), for example, respiratory technique, oral health, incontinence, sexual issues, relationships, and cohabitation. In addition to lectures, the participants are shown various self‐care techniques and allowed to practice, which is appreciated; “I wish I had much more of that” (P9). The fact that there is no cure for the disease creates concern for the future and a wish to discuss this topic further.

Lectures and discussions are combined with physical examinations and breathability tests. This is described as providing a sense of security for future activities, such as before a flight. The participants describe the initial examination and tests by the nurse as a good preparation for the group sessions. There is a need to have an opportunity to ask the nurse personal questions at the end of the group sessions.

The focus on lectures during group meetings is sometimes experienced as too specific and participants feel that there is no room for more general questions: “They were very specialised” (P9). When the content becomes too specific, there is a risk that it is not applicable to all participants: “The information contains elements that I may not feel I need” (P4). However, there is an awareness that the participants have different needs and conditions and are in different stages of the disease. The fact that the lectures are combined with COPD information on the website enables the search for relevant information according to participants.

Formal learning in the form of teaching and exercises is combined with informal learning. The informal learning takes place during breaks and is described by the participants as very rewarding. This is when experiences and advice are shared, and general information can be understood from a personal perspective. More time and space are requested for this, which is expressed in the following way: “There is a lot I would like to ask about…. my mornings are crap, I can say” (P10). The combination of different teaching methods is appreciated, especially the sharing of experiences.

### 3.2 Sharing experiences – Reduces the feeling of being alone

For persons with COPD, group‐based self‐care education with digital support means a shared experience that reduces the feeling of being alone and supports learning. Sharing experiences is possible when the meetings take place regularly and with persons living with COPD. When the experiences of living with COPD and the feelings related to the illness and treatment, thoughts about the future and feelings such as shame and guilt are shared, the feeling of loneliness is reduced. This is expressed as: “Safe to know that you are not alone” (P6). Even though there is an awareness that it is not the same for everyone: “They may feel different” (GP1), sharing experiences contributes to learning as it is even difficult to talk about feelings related to the illness with relatives. Participants express a sense of guilt that they themselves have caused the disease: “There may be this feeling – well, you smoked” (P13).

It is considered easier to listen and talk to persons with similar experiences: “You learn more when you are in a group” (GP1). To share experiences, one must put into words one's own experiences and questions: “You remember what you talk about yourself better than what you just listen to” (GP1). Sharing also means that the participants get answers to questions that they themselves do not want to address: “…it is positive when you are in a group and others ask questions and I ask questions you wonder about different things and then you get to know more, I think” (P8).

Participants with a sense of humour and a positive attitude help create a positive environment that is described as relieving and important to achieving a learning climate. However, the sharing of experiences is limited. Serious concerns about the future such as fears of slowly suffocating and dying are described and the fact that the prognosis is bad gives no hope of improvement: “It creates a lot of anxiety… I almost lose the zest for life some days” (P7). Despite the sharing of experiences, the participants believe that the group refrains from talking about existential issues: “perhaps they avoid talking about the worst” (P7). Hence, professional support to deepen the conversations is requested. As the prognosis of the disease is dismal, more hope for the future is sought.

The opportunity to bring relatives to the group meetings is perceived as valuable by both participants and relatives: “It's nice that they got to know not only from me” (P3), but the schedule for the group meetings makes it difficult. When this is possible, a common knowledge is created that also contributes to an increased understanding of how the illness affects those living with COPD. Involving close relatives in the group sessions contributes to a sharing of information and responsibility over what has been discussed in the group. This sharing is also made possible by the COPD website to which the participants sometimes refer relatives. However, sharing with relatives is limited as patients do not want to burden their relatives with their illness, thoughts, and feelings. The sharing is described as statistically significant and something that the participants need over time: “I probably would have liked to continue with the group” (P3). Sharing experiences over time creates opportunities to reduce the feeling of being alone resulting from the disease.

### 3.3 Changed understanding – As a result of learning

For persons with COPD, group‐based self‐care education with digital support entails a changed understanding, that is, a learning that provides the drive and courage to carry out various projects. The knowledge and understanding of illness, its treatment, and self‐care changes during the COPD school, and the new changed understanding enables a more active life. According to a participant, before COPD school there was a fear of exercises worsening their illness. However, increased knowledge and understanding of the importance of physical activity led to increased activity and consequently improved well‐being. Another participant expresses it as: “I have now gained insight that I should do breathing exercises and other exercise” (P8). The changed understanding can also lead to increased awareness of the limitations posed by the disease. Furthermore, it provides insight of the significance of the disease in daily life, both for the person with COPD and their next of kin. A married man talks about increased insight into his wife's situation, that he sometimes pressured her too much, for example, when walking: “I may have stressed her too much and told her that she does not keep up, so I have learned a lot” (GP2). Thus, changed understanding can lead to a new common perspective on the situation.

A changed understanding is also described as insights that relieve feelings of guilt and shame. Group participants describe a relief in knowing that COPD also affects persons who do not smoke or have never smoked. Another insight is that the disease also affects younger people. Further insights are that it is okay to rest when energy runs out. The participants get a lot of tips that improve their everyday life from the group meetings and the COPD website, such as “repositioning in the kitchen cabinets, by moving things often used to the lower shelves” (P5) or having: “ready‐made food in the freezer” (P4). There is an increased understanding of the effect of medicines, how they interact with each other, why they should be taken in a certain order, and how they should be administered for best effect. The changed understanding creates power of action but also provides insight into the limitations in life that the disease entails, limitations that persons with COPD and their relatives must adhere to.

### 3.4 Continuous learning – Opportunities and obstacles

Group‐based self‐care education with digital support provides opportunities for continuous learning, unique support for rehearsal, in‐depth studies and information about the disease and its treatment for patients with COPD. The website is described as easy to search and find information on, and the films are described as illustrative. The option of being able to look up a question several times if needed gives a feeling of safety. The website is described as comprehensive: “a goldmine to scoop out” (GP2) and “you get tips on different things” (GP2). At the same time, there is a risk of the learning process failing if the understanding is not challenged in interaction with others, for example, within group‐based education. The participants describe the group education as the start of their learning process: “Because this starts a lot of thoughts, and it raises questions” (GP1) of importance for learning to live with COPD. The ongoing learning evokes curiosity which further encourages the participants to read more on the COPD website. Some do not use the website, instead they prefer to search via Google but must then be careful to evaluate the sources. Others have used the website before the group sessions and now use it frequently after the group sessions. These are mainly persons with IT skills. According to a participant, she found out that she had COPD through the COPD website.

The opportunity of following up on the health condition with a nurse at the hospital is experienced as valuable for those who have it and lacked by those who do not have it. This follow‐up is described as important in getting answers about the prognosis of the disease over time. It is also valuable in addressing questions that arise in life with COPD. The group‐based self‐care education with digital support has also encouraged patients to seek support from a physiotherapist to start training, keep it up, and get advice. But even here, the COPD website seems to be a support: “I see these movements as good and then you can have the programme on and then you can sit and do exactly as they do. So, I think that's good” (P4).

Thus, the follow‐up contacts and the COPD website support continuous learning in living with COPD.

It appears that the participants choose not to introduce next of kin to the COPD website so as to not burden them: “I try not to burden persons close to me with my worries, so I try to keep it to myself” (P7).

This can be understood as an obstacle to continuous learning. On the other hand, the COPD website is seen as an opportunity for next of kin to familiarise themselves with the situation: “Important for persons close to me, they do not understand” (P13).

The COPD website is, however, limited to those who have technical opportunities and competence. A resistance is described as: “going into the computer and read if you are not used to read online” (GP1), the texts can be perceived as too long, and it can then be difficult to see the benefit of reading online. On the other hand, travelling to attend the group sessions is described as stressful and takes a lot of time, so the COPD website is a good complement to continuous learning.

## 4 DISCUSSION

The result of this study corresponds with the visions of future healthcare and highlights the need for both person‐centred care and for eHealth (McCarthy et al., 2015; The National Board of Health and Welfare, 2018; World Health Organization, 2020), which has the potential to facilitate sustainable learning that supports patients' self‐management. Research has shown that people with asthma/COPD value advice on managing symptoms as support for self‐management over time (O'Connell et al., 2021). The findings in this study show that the text and the films on the COPD website provide opportunities to gain information before, during, and after the group sessions and contribute to continued learning both for the patients and their family members. This may be seen as effective in improving the accessibility and safety of healthcare in patients with COPD and other chronic conditions. The result shows that a combination of teaching methods in group settings and on the website is valued to support learning to live with COPD. As Kolb (1984) describe people have different learning styles (active, reflective, logical, and practical), it is important to support learning based on these styles. The content in the group sessions and the information on the COPD website cover a wide range of interests and information needs. However, there is a risk that a wide range of content in group education settings may reduce time for sharing experiences and reflections over existential issues, that is, what it means to me as a person and how I can use this knowledge in daily practice. Other studies have highlighted the importance of deepening the learning in a way that involves the participants on both an emotional and a concrete practical level. By giving the time to share and reflect over experiences with the group members (Anderson et al., 2010; Kjellsdotter et al., 2021), the feeling of not being alone may be supported. Furthermore, the essential meaning of learning to live with a long‐term illness concerns existential issues such as having a reduced access to the daily life. In this learning process, in which everyday lives as well as relationships with oneself and others are affected, an ongoing renegotiation needs to be supported. Sustainable learning can form a life‐world theoretical perspective where the person deeply reflects about their life, spirituality, and feels a responsibility to make decisions that are important for them as a person with a disease, described as a genuine learning (Berglund & Källerwald, 2012). As the results show, it is important that an opportunity is given for in‐depth reflection on how the disease affects personal life instead of just a general perspective. This perspective on learning can also be understood in relation to “effective learning” and Kolbs theory (Kolb, 1984), where it is described as learning in cycles. The process starts in a concrete experience followed by observation, reflection, and analysis, where the hypothesis is tested and then used in the future as the basis of a new experience. We argue that these aspects of learning must be supported if the aim is to reach sustainable learning. Self‐management theories and learning perspectives should influence the digital design and development (Sangrar et al., 2019), especially when education is increasingly imparted digitally. In addition, our result corresponds with the need for both physical group sessions and the information on the website.

The results indicate that group‐based education sessions are a valuable arena to reflect together as a group that the website in its current form does not provide. However, from a professional perspective, this requires an awareness of a tactful didactic approach which supports and challenges comprehension as well as action (Andersson et al., 2019; Berglund & Källerwald, 2012). The results of the current study illustrate that group sessions create opportunities for learning when group members address questions that open up for a new understanding of life with the illness. In total, the website and the group education contribute to a decreased feeling of loneliness in their current situation. Thus, there is a potential for improvement regarding opportunities for important reflections over lived experiences and feelings such as shame and guilt related to the disease. Furthermore, the current study and other research highlight that learning to live with a long‐term illness needs support over time and give prerequisites for self‐management and a sense of well‐being (Kruis et al., 2013; Stoilkova‐Hartmann et al., 2018).

The digital website presents an opportunity to continuously learn to update and maintain previous knowledge and seek out new information when the need arises. Patients and relatives can easily access this information regardless of the time. In the future, digital resources might create time and space for reflective conversations on the COPD website with virtual chatrooms. On the other hand, digital solutions require technical equipment, access to network, and support when needed.

### 4.1 Strength and limitations

The lived experience in RLR is a suitable method to give a deeper understanding of the studied phenomenon (Dahlberg et al., 2008). The phenomenon in this study, group‐based self‐management education with a digital website was studied by data collected through interviews. A strength of the study was that the interviews were conducted both in groups and individually. During the interviews, the atmosphere was open and everyone was given the opportunity to speak. The data contained rich and detailed information as a result. RLR required an open and reflective approach regarding the understanding of the phenomenon during the research process, demanding the researcher to be bridling in relation to the phenomenon to ensure validity (Dahlberg & Dahlberg, 2020). The transferability of the findings was possible through a rich variation of experiences, which according to Dahlberg and Dahlberg (2020) potentially contributed to the results' generalisability and transfer to similar groups of patients with long‐term illnesses and high self‐care demands. As with all qualitative studies, the transferability of our results to a similar context and to other countries must be valued and assessed by the reader. This study was performed in a Swedish context, which might be a limitation.

## 5 CONCLUSION

The group‐based self‐management education with a digital website was developed by patients with experiences of COPD together with a multidisciplinary team. A combination of both physically and digitally group‐based self‐management education has demonstrated that adapting learning activities to individual learning styles increases sustainability of learning. Sharing experiences reduces feelings of loneliness. It is therefore important to create spaces for sharing experiences and in‐depth reflection that support learning. For learning to be sustainable for patients with long‐term illness, continuous learning based on experience and knowledge is needed, which requires support from health professionals.

## 6 RELEVANCE TO CLINICAL PRACTICE

It is important to create space, both physical and digital, for patients with COPD to share experiences and reflect together. The reflection can be supported and challenged by health professionals to become genuine and support sustainable learning. The health professionals need to increase patient involvement when creating future self‐management education programmes, which in turn implies a need to change routines and establish new ways of organising and administering care. The new knowledge can be useful in developing person‐centred care. The health professionals need education and support in this new approach.

## FUNDING INFORMATION

We would like to thank the Research and Development Centre at Skaraborg Hospital Skövde, Sweden and the Skaraborg Institute, Sweden for their support (Dnr. 19/1034).

## CONFLICT OF INTEREST STATEMENT

The authors have no conflicts of interest to declare and certified that they have seen and approved the manuscript.

## Supporting information


Appendix S1.

## Data Availability

Research data are not shared due to the personal nature of the interview data collected and the possibility that participants could be identified.
